# Prevalence of vertical HIV infection and its risk factors among HIV exposed infants in East Africa: a systematic review and meta-analysis

**DOI:** 10.1186/s41182-020-00273-0

**Published:** 2020-10-20

**Authors:** Amare Belachew, Tilahun Tewabe, Gizat Abinet Malede

**Affiliations:** 1 Department of Pediatrics and Child Health Nursing, College of Medicine and Health Sciences, Bahir Dar University, Bahir Dar, Ethiopia; 2 Department of Laboratory Science, Bahir Dar Health Science College, Bahir Dar, Ethiopia

**Keywords:** HIV, Incidence, MTCT, PMTCT, Prevalence, Burden, Risk, Vertical HIV infection, Rate of vertical HIV infection, HIV-exposed infant, East Africa

## Abstract

**Background:**

Human immunodeficiency virus (HIV) is one of the most important global health problems. More than one and half million of children are living with HIV in the world, and majority of them are found in sub-Saharan Africa. There are primary fragmented study findings, and no review was conducted with regard to vertical HIV infection in East Africa. Therefore, this review aimed to assess the prevalence of vertical HIV infection and its risk factors among HIV-exposed infants in East Africa.

**Main body:**

Eligible studies were retrieved by relevant search terms in CINHAL, Pub-MED, Google Scholar, EMBASE, Web of Science, SCOPUS, Cochrane, African Journals Online databases, and Ethiopian University research repositories. Data were extracted with Microsoft Excel and analyzed with Stata version 11 software. The random effect model was used to estimate the pooled prevalence of vertical HIV infection in East Africa. The variation between studies was quantified with an *I*^*2*^ statistic test. Furthermore, sub-group and meta-regression analyses were done to identify the sources of heterogeneity between the studies. The publication bias was assessed by Egger test. This systematic review and meta-analysis have included a total of 33 research articles. The overall pooled prevalence of vertical HIV infection in East Africa was 7.68% with a 95% confidence interval [CI]: (6.23, 9.12) with a heterogeneity of *I*^2^ = 86.8 with a *p* value < 0.001. In subgroup analysis, the pooled prevalence of vertical HIV infection in cross-sectional studies was 6.58%, while in cohort studies were 9.37%. Mixed feeding, AOR = 6.22 (1.02, 11.41); home delivery, AOR = 2 (1.01, 3); mothers took ART less than 4 weeks, AOR = 1.92 (1.79, 2.06); and infants who have not received ARV prophylaxis, AOR = 2.02 (1.05, 2.98) were the associated factors for vertical HIV infection for exposed infants.

**Conclusions:**

The pooled prevalence of the mother to child transmission of HIV is way more than the desired target of the World Health Organization, which is less than 5% in breastfeeding populations. Thus, strengthening the prevention of vertical HIV transmission, promotion of exclusive breastfeeding, timely initiation of ART prophylaxis for HIV exposed infants, encouragement of hospital delivery, and the start of ART at the time of diagnosis of every HIV-positive person may all reduce the transmission of vertical HIV infection.

## Background

Human immunodeficiency virus (HIV) affects our immune system that helps to defend infections and illnesses in our body system [1]. About 37.9 million people are the carrier of HIV in the globe. Of this, 1.7 million are children and most of them are found in sub-Saharan Africa [1].

The youngsters were acquired HIV during pregnancy, childbirth, and breastfeeding [1, 2]. The chance of vertical HIV infection without any intervention during pregnancy and delivery reach to 15–30% [2], breastfeeding, 5–20% [3], and overall transmission reach to 15–45% [3, 4]. Providing timely antiretroviral prophylaxis for HIV-exposed infants and ART for HIV-positive mothers are helping to cut back the risk below 5% [3, 4].

Different strategies were recommended and implemented by WHO to prevent vertical HIV infection in different countries such as increasing antenatal care visits, promoting institutional delivery, strengthening prevention of vertical HIV transmission services, and ART services [1, 3, 4]. But the problem is still prevalent in sub-Saharan Africa countries [1].

Different studies were conducted to assess the magnitude of vertical HIV infection, and its association with mixed feeding, place of delivery, timely initiation of ART prophylaxis among HIV-exposed infants, and duration and initiation of antiretroviral therapy (ART) for HIV-infected mothers in East Africa [5–37]. But the reports of these research findings brought with great differences within the magnitude of vertical HIV infection across the countries’ geographical regions.

The place of delivery [6, 7, 10, 14, 18, 21, 24, 25, 27, 38, 39], mixed feeding [6, 9, 15, 21, 25, 27, 39], mother’s age [5, 24], residence [7, 27], time to initiate infant prophylaxis [7–10, 27], mother’s HIV stage [9, 18], antenatal care visits [8, 9, 18], prevention of vertical HIV infection [6, 7, 24], and feeding practices [7, 8, 24] were a number of the factors for acquisitions of HIV infection for exposed infants and children. Of these factors, four factors (place of delivery, duration of mother on ART, initiation of ART prophylaxis for newborns, and mixed feeding) were chosen to work out their effect on vertical HIV infection. Within the bulk of studies, the chosen factors were predictors of vertical HIV infection, while in some studies, they showed a negative association. This disagreement is not well investigated. Thus, the purpose of this review aimed to estimate the pooled prevalence of vertical HIV transmission and its associated factors among HIV-exposed infants in the geographical regions. This study finding may help an input for program planners, used to monitor the progress of PMTCT programs, policymakers, and further research.

## Methods

This systematic review and meta-analysis followed the guideline of Preferred Reporting Item for Systematic Reviews and Meta-analysis [40]. The protocol of the development of this systematic review and meta-analysis is under process on PROSPERO databases with ID of 147647.

### Search strategy and study design

Eligible published studies were searched in CINHAL, Pub-MED, Google Scholar, EMBASE, Web of Science, SCOPUS, Cochrane, African Journals Online databases, and Ethiopian University research repositories. The searching terms were “prevalence”; “burden”; “ proportion”; “incidence”; “factors”; “barriers”; “predictors”; “vertical HIV infection”, “HIV exposed infants”, “mother to child”; “mother to baby”; “mother”; “infant”, ‘MTCT’; “ Mother to child transmission of HIV”, “HIV”; “human immunodeficiency virus”; “AIDS”; “Acquired immunodeficiency syndrome”; and “East Africa or each specific countries (Ethiopia, Kenya, Tanzania, Djibouti, Eritrea, Uganda, Somali, South Sudan, Rwanda, Zimbabwe, Mozambique, Malawi, and Burundi )”. Boolean operator terms were searched separately and together accordingly as “OR” or “AND” or AND NOT or AND, NOT. Gray literature from the research online library repository and manual search of references were also searched.

### Eligibility criteria

Studies were included if: (1) studies conducted in East Africa; (2) all studies were done regarding the prevalence of vertical HIV infection or its association with mixed feeding, place of delivery, time of prophylaxis begun, and length of starting ART drug among HIV-exposed infants in East Africa; (3) papers published only in the English language. Studies were excluded in this meta-analysis when: (1) the study is qualitative, (2) was published in a book, (3) research reports that were not accessed and not written in English were excluded because of difficulty in assessing the quality of each article.

### Measuring outcome variables

This review has two outcome variables. The first outcome of this review was the magnitude of vertical HIV infection among HIV-exposed infants. The HIV-positive infant was confirmed at 6 weeks or after using deoxyribonucleic acid-polymerase chain reaction (DNA-PCR) virology tests or 18 months or above using the DNA-PCR test or rapid antibody test after 6 weeks of breastfeeding cessation [4]. The duration of the study ranges from birth to 24 months old. The second outcome was to look at the association between mixed feeding, duration of mother on ART, infant ART prophylaxis at birth, place of birth, and vertical HIV infection. In this study, mixed feeding implies that HIV-positive mothers feed their newborn infant both breast milk and other food or fluid within 6 months of birth. The adjusted odds ratio was undertaken between mixed feeding, initiation of ART prophylaxis, duration of mother on ART, place of birth, and vertical HIV infection of infants.

### Quality assessment

Each research article was critically appraised by using the Joanna Briggs Institute Meta-Analysis of Statistics Assessment and Review Instrument (JBI-MAStARI) [41]. The criteria include both the inclusion and clear definition criteria of the inclusion of sample, selection of the study participants, confounding factors, outcome of interest, and consistent measurement of outcome variable and statistical analysis methods [41]. Reviewers were dealing it before the extraction of the data. Two independent reviewers carried out the searches to extract the data. Data extraction was carried out by the Joanna Briggs Institute (JBI) tool for prevalence studies [41]. It includes a primary author, year of the publication, study area, sample size, study design, region of the study, prevalence of vertical HIV infection, time of infant HIV diagnosis, and a total number of HIV-positive and HIV-negative infants by breastfeeding option, infant ARV prophylaxis, place of delivery, and mother’s PMTCT intervention status. Reviewers independently carried out article inclusion, data extraction, and compared results. Finally, the disagreements were resolved by a consensus between the two reviewers.

### Statistical methods and analysis

STATA version 11 software was used to analysis of the data. The pooled magnitude of mother to child transmission of HIV was estimated with DerSimonian and Laird’s random-effects model. Furthermore, subgroup and univariate meta-regression analyses were undertaken with sample size, study setting, study design, country, and publication date. The adjusted odds ratio was used to predict between vertical HIV infection with mixed feeding, place of delivery, duration of mother on ART, and infant received ART prophylaxis at birth.

### Heterogeneity and publication bias

The *I*^2^ statistic test was used to quantify heterogeneity between the studies and a *p* value less than 0.05 declared as statically significant [42]. The Duval and Tweedie nonparametric trim and fill analysis followed by Egger test were conducted for assessing publication bias (Egger test, *p* < 0.05) [41–43].

## Results

**Fig. 1:**
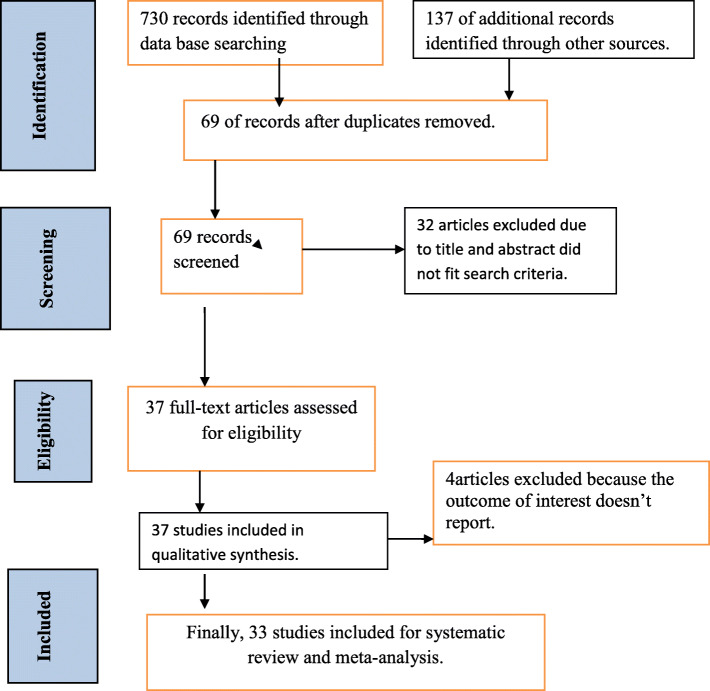
Flowchart diagram of search strategy and article selection for vertical HIV infection in East Africa, 2019

A total of 867 articles were retrieved regarding vertical HIV infection in East Africa. Of this, 415 were searched from PubMed, 225 articles from Google Scholar, 90 articles from ScienceDirect, and 137 from others. From total extracted articles, 798 were expelled because of duplications and irrelevancies, and 32 were removed after abstract and title screening. But thirty-seven studies met the eligibility criteria, and four articles were removed because they did not report the outcome interest [44–47]. Finally, 33 studies were included in this meta-analysis (Fig. 1).

### Characteristics of the included studies

A total of 33 published articles were included in this study. In this review, a total of 428124 of HIV exposed infants were involved to calculate the pooled magnitude of vertical HIV infection. Articles done both on cohort and cross-sectional study designs were incorporated in this study. The peak prevalence (32.1%) of MTCT of HIV was found from a study done in Ethiopia [28], while the lowest magnitude (1.58%) was found in Rwanda [36]. A total of nine countries and 33 studies were included in this systematic review and meta-analysis. Of this, sixteen studies were from Ethiopia (5-11, 21-24, 26-28, 37, 38 ), five studies were from Kenya [12, 15, 20], three studies were from Tanzania [13, 16], three studies were from Uganda [14, 18, 19, 31], two studies were from Zimbabwe [33, 34], and the remaining one study was from Eritrea [19], South Sudan [32], Malawi [35], and Rewanda [36]. In this review, eighteen studies were cross-sectional study designs while fifteen studies were cohorts (Table 1).

**Table 1:** Descriptive summary of 33 studies included in the meta-analysis of the prevalence of vertical HIV infection in East Africa, 2019

Authors/year of publication	Country	Study design	Sample size (*N*)	Magnitude of vertical HIV infection (%)	Quality
Moges et al. 2017 [5]	Ethiopia	Cohort	305	5.9	Low risk
Obsa et al. 2018 [6]	Ethiopia	Cohort	492	7.7	Low risk
Birlie et al. 2016 [7]	Ethiopia	Cohort	146	17	Low risk
Derbe et al. 2014 [8]	Ethiopia	Cohort	426	9.6	Low risk
Tadele et al. 2014 [9]	Ethiopia	Cohort	457	4.16	Low risk
Wudineh et al. 2016 [10]	Ethiopia	Cohort	382	15.7	Low risk
Negash et al. 2016 [11]	Ethiopia	Cohort	384	6	Low risk
Sirengo et al. 2014 [12]	Kenya	Cross-sectional	63	12.7	Low risk
Gamell et al. 2017 [13]	Tanzania	Cohort	135	2.2	Low risk
Kahungn et al. 2018 [14]	Uganda	Cross-sectional	493	6.5	Low risk
Mwau et al. 2017 [15]	Kenya	Cross-sectional	365841	8.9	Low risk
Mwando et al. 2014 [16]	Tanzania	Cross-sectional	561	9.6	Low risk
Mirambo et al. 2014 [17]	Tanzania	Cross-sectional	1005	6.1	Low risk
Izudi et al. 2018 [18]	Uganda	Cohort	295	8.5	Low risk
Teclebirhan et al. 2009 [19]	Eritrea	Cohort	105	4.7	Low risk
Khobondo et al. 2015 [20]	Kenya	Cross-sectional	360	10.6	Low risk
Tsehay et al. 2019 [21]	Ethiopia	Cross-sectional	27	5.8	Low risk
Desta [22]	Ethiopia	Cross-sectional	350	2.1	Low risk
Mirkuzie et al. 2010 [23]	Ethiopia	Cross-sectional	896	11.8	Low risk
Koye et al. 2013 [24]	Ethiopia	Cohort	509	10	Low risk
*MAMA A et al. 2015* [25]	Ethiopia	Cross-sectional	10	7.7	Low risk
Mirkuzie et al. 2011 [26]	Ethiopia	Cohort	71	8.4	Low risk
Berhan et al. 2014 [27]	Ethiopia	Cohort	434	10.10	Low risk
Hassen et al. 2014 [28]	Ethiopia	Cohort	159	32.1	Low risk
Makau et al. 2015 [29]	Kenya	Cross-sectional	238	8.4	Low risk
Ashino et al. 2017 [30]	Kenya	Cross-sectional	2642	9.27	Low risk
Obai et al. 2017 [31]	Uganda	Cohort	410	5.8	Low risk
Gawar (a) et al. 2019 [32]	South Sudan	Cross-sectional	828	2.8	Low risk
Buzdugan etal. 2015 [33]	Zimbabwe	Cross-sectional	8568	8.8	Low risk
Ndaimani et al. 2018 [34]	Zimbabwe	Cross-sectional	5836	4.4	Low risk
van Lettow et al. 2018 [35]	Malawi	Cross-sectional	33744	4.7	Low risk
Mugwaneza et al. 2018 [36]	Rewanda	Cross-sectional	1639	1.58	Low risk
Yitayew [37]	Ethiopia	Cross-sectional	313	3.8	Low risk

### Publication bias

**Fig. 2:**
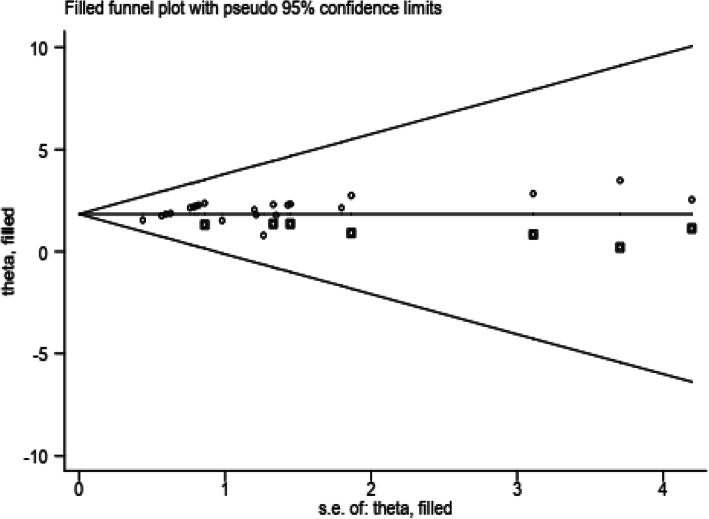
Frim-trill analysis result of 33 included studies for vertical HIV infection in East Africa, 2019

Publication bias was assessed by Egger test. The result of Egger test was statistically significant for estimating the magnitude of HIV-positive infants (*p* < 0.001). Egger test of the intercepts (B0) was 0.99 ((CI) =0.973, 1.02). Trim fill analysis was conducted and it showed that there is a remaining study in graphs. But the confidence interval is overlapped and it is not significant (Fig. 2).

### Overall pooled magnitude of vertical HIV infection

**Fig. 3:**
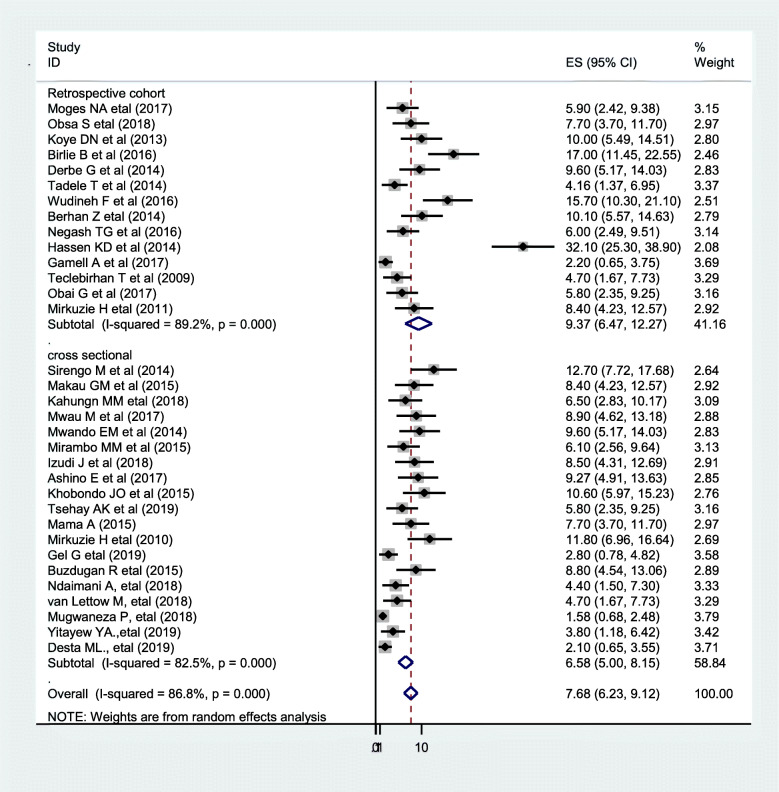
Forest plot of the prevalence with corresponding 95% CIs of sub-group analysis by the study design of the twenty-seven studies on vertical HIV infection in East Africa. The midpoint and the length of each segment indicated prevalence and a 95% CI whereas the diamond shape showed the combined prevalence of all studies

The result of thirty-three studies showed that the pooled magnitude of vertical HIV infection in Eastern Africa was 7.68% (CI 6.23, 9.12). There was a severe heterogeneity across the studies (*I*^2^_=_ 86.8%, *p* value < 0.001). Due to severe heterogeneity, subgroup analysis was carried out with study design (Fig. 3). The higher prevalence of vertical HIV infection was observed in Ethiopia (32.1%), while the lower prevalence was in Rwanda (1.58%).

### Meta regressions

The univariate meta-regression model was done with publication date, sample size, country, and study design for identifying the source of heterogeneity and none of them were statically significant (Table 2).

**Table 2:** Univariate meta-regression analysis for vertical HIV infection in East Africa, 2019

		Co-efficient	*p* value
Year of study		− 0.225	0.696
Sample size		0.000	0.674
Countries	Ethiopia	5.9	1
	Kenya	.232	0.8
	Tanzania	.329	0.702
	Uganda	0.133	0.847
	Eritrea	1	
Study design	Cross-sectional	− 0.232	0.726
	Cohort	1	

### Place of delivery and vertical HIV infection

**Fig. 4:**
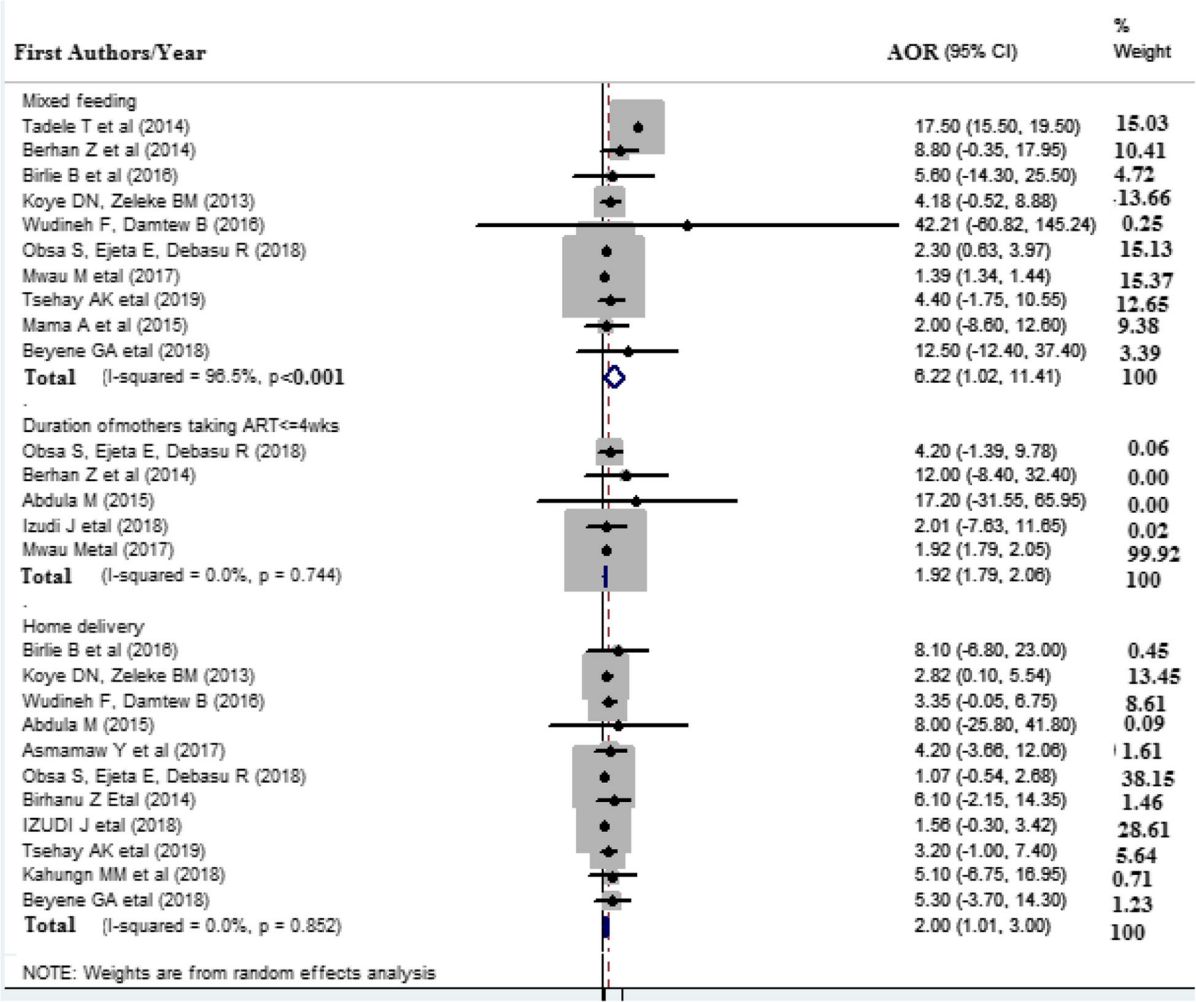
Forest plot displaying the association of selected factors with vertical HIV infection among exposed infants in East Africa. The midpoint and the length of each segment indicated an AOR and a 95% CI, the arrow showed the widest CI, and the diamond shape showed the combined AOR of all studies for each variable

Eleven studies were included to assess the association between place of delivery and HIV infection [6, 7, 10, 14, 18, 21, 24, 25, 27, 37, 38]. The findings showed that the place of delivery was significantly associated with vertical transmission of HIV. The odds ratio of infants who were delivered at home were 2 times more likely to acquire HIV infection than those who were delivered at health facilities (AOR: 2.00, 95% CI 1.01, 3.00). No heterogeneity (*I* = 0.0% and *p* value = 0.852) was observed across the included studies (Fig. 4).

### Mixed feeding and vertical HIV infection

A total of ten studies [6, 7, 9, 10, 15, 21, 25, 27, 38] were included in this meta-analysis to show the significance between mixed feeding and MTCT of HIV. The pooled odds ratio showed that mothers practiced mixed feeding for their exposed infants were 6.22 times more likely to acquire HIV infection than exclusively breastfeeding exposed infants (AOR = 6.22 (1.02, 11.41). There is a severe heterogeneity across the studies (*I*^2^ = 96.5% and *p* value < 0.001). Egger test was done to detect the publication bias, but there is no revealed significant on publication bias with a *p* value of 0.074 (Fig. 4).

### Duration of mothers on ART and MTCT of HIV

Five studies ( [6, 15, 18, 25, 27]) were included in this study to estimate the pooled effect of the duration of the mother’s on ART with vertical HIV infection. The odds ratio of mothers taking ART less than 4 weeks were 1.92 times more likely to transmit HIV infection to their exposed infant as compared to those who were on ART for more than 4 weeks (AOR = 1.92 (1.79, 2.06). No heterogeneity (*I* = 0.0% and *p* value = 0.744) was observed across the included studies (Fig. 4).

### Absence of infant received ART prophylaxis at birth and MTCT of HIV

**Fig. 5:**
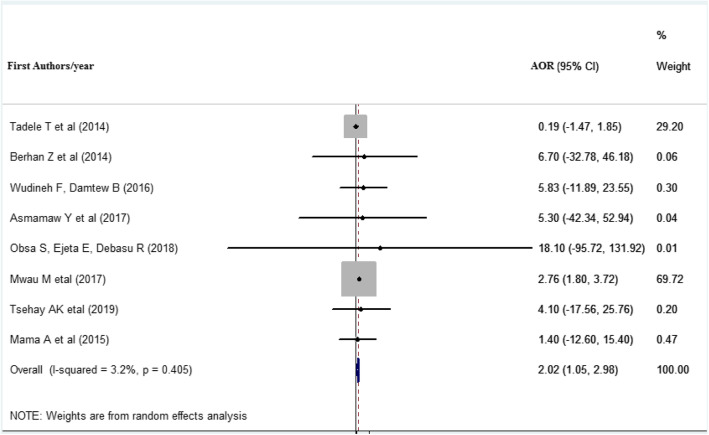
Forest plot displaying the association of infants who were not received ART prophylaxis with vertical HIV infections among exposed infants in East Africa. The midpoint and the length of each segment indicated an AOR and a 95% CI, the arrow showed the widest CI, and the diamond shape showed the combined AOR of all studies for each variable

Eight studies ( [6, 9, 10, 15, 21, 25, 27, 38]) included in this meta-analysis to indicate the impact of providing ART prophylaxis at birth. Infants who were not taking ART prophylaxis were 2.02 times more likely to acquire HIV infection than those who were taking ART prophylaxis (AOR = 2.02 (1.05, 2.98). The heterogeneity across the study was (*I*^2^ = 3.2% and *p* value = 0.405) (Fig. 5).

## Discussion

While much effort was done to tackle the vertical transmission of HIV in East Africa, the transmission of mother to child is still prevalent. Thus, this systematic review aimed to assess the pooled prevalence of vertical HIV infection and its associated factors among HIV-exposed infants in East Africa.

The overall pooled prevalence of vertical HIV infection among HIV-exposed infants in East Africa was 7.68% (95% CI 6.23, 9.12), and this finding is high compared to WHO setting strategies of achieving zero incidences of new HIV infection among HIV-exposed infants by 2020 [48, 49]. This finding is higher than studies done in China, 3.9% (95% CI 3.2, 4.6%) [49], and Cuba and Thailand eliminated of MTCT of HIV [50, 51]. The possible reason for this discrepancy may be there is deference’s in socio-demographic characteristics, economic, access to ART services, and health care-seeking behavior of the respondents. Additionally, countries like Cuba and Thailand have maintained a strong commitment to the PMTCT of HIV and successfully integrated PMTCT interventions into maternal and child health services than East Africa countries [50]. Similarly, this finding also shows the high-vertical transmission of HIV compared to South Africa, the risk of MTCT at 18 months was 4.3% [52]. This discrepancy may be due to in South Africa, more than 95% antenatal HIV testing uptake and triple ART coverage (≥ 93%) compared to the current study settings and this helps in the reduction of MTCT of HIV in South Africa compared to this study [52]. Additionally, there is a literature report that there is a poor intake of PMTCT services in developing countries due to different reasons as compared to the developed ones [53].

The sub-group analysis of both cohort and cross-sectional studies has a similar pooled prevalence of vertical HIV infections, which accounts for 9.37% and 6.58%, respectively. While they have similar pooled prevalence, there was observed high heterogeneity in cohort studies than cross-sectional ones.

Vertical HIV infection was influenced by various factors. Infants who delivered at home were 2.00 times to acquire HIV infection compared to those who delivered at health facilities. This finding is in line with studies done in Nigeria [53] and Zimbabwe [54]. This might flow from to inaccessible of PMTCT services for those who were delivered at home. Besides, mothers who gave birth at home may have a high probability to practice prelactal feeding, poorly managed cords like using unsterilized sharp materials, high chance of placenta contamination with the blood, breastfeeding of infants from the unknown status of mothers, and uvulectomy practices. These activities may expose newborns more for viral infections [55–58].

Mixed feeding is one of the most risk factors of acquiring HIV infection for exposed infants. HIV-exposed infants who fed both breast milk and other liquid or fluid within the 6 months of birth were 6.22 times more acquired HIV infection compared to exclusive breastfeeding infants. The result of this finding is consistent with the study findings in Zimbabwe [58] and South Africa [59]. This may be due to mixed feeding cause gastric mucosal ulceration and the laceration of mucus creates a favorable entry of the virus into the bloodstream [4, 60].

Infants who did not receive ART prophylaxis at birth were 2.02 times more likely to acquire HIV infection compared to those who were taken ART prophylaxis at birth. Similarly, mothers who took ART drugs less than 4 weeks were 1.92 times more likely to transmit HIV infection than those who were taking more than 4 weeks. The finding of this study result was agreed with studies done in French [61], Africa [62], and UK [63]. This is because early initiation of ART to the mother during pregnancy period plus prophylaxis of ART for HIV-exposed infants at birth decreases the viral load of the virus and reducing the chance of vertical HIV infections of newborns.

## Conclusions

Generally, the pooled prevalence of vertical transmission of HIV is high in the study area compared to other studies. Thus, strengthening the prevention of mother to child transmission of HIV (PMTCT) program, promote exclusive breastfeeding, timely initiate ART prophylaxis for HIV exposed infants, and encourage hospital delivery, and timely initiation of ART drugs for mothers were recommended. It is the first meta-analysis carried out in East Africa. Only English articles were considered to carry out the analysis. Studies in this review were included only cross-sectional and cohort in nature, and therefore, the outcome variable might be affected by other confounding variables. The estimated report could be affected by the limited sample size.

## Data Availability

Data will be available upon request of the corresponding author.

## Abbreviations

ANC: Antenatal care
CI: Confidence interval
EDHS: Ethiopian Demographic and Health Survey
HIV: Human immune deficiency virus
OR: Odds ratio
MTCT: Mother to child transmission
PMTCT: Prevention of mother to child transmission
WHO: World Health Organization
